# The Future of Robotic Surgery in Pediatric Urology: Upcoming Technology and Evolution Within the Field

**DOI:** 10.3389/fped.2019.00259

**Published:** 2019-07-02

**Authors:** Kunj R. Sheth, Chester J. Koh

**Affiliations:** ^1^Division of Urology, Department of Surgery, Texas Children's Hospital, Houston, TX, United States; ^2^Scott Department of Urology, Baylor College of Medicine, Houston, TX, United States

**Keywords:** robotic, laparoscopic, pyeloplasty, heminephrectomy, ureteroureterostomy, children, pediatric, urology

## Abstract

Since the introduction of the Da Vinci Surgical System (Intuitive Surgical, Inc., Sunnyvale, CA) in 1999, the market for robot assisted laparoscopic surgery has grown with urology. The initial surgical advantage seen in adults was for robotic prostatectomy, and over time this expanded to the pediatric population with robotic pyeloplasty. The introduction of three-dimensional visualization, tremor elimination, a 4th arm, and 7-degree range of motion allowed a significant operator advantage over laparoscopy, especially for anastomotic suturing. After starting with pyeloplasty, the use of robotic technology with pediatric urology has expanded to include ureteral reimplantation and even more complex reconstructive procedures, such as enterocystoplasty, appendicovesicostomy, and bladder neck reconstruction. However, limitations of the Da Vinci Surgical Systems still exist despite its continued technological advances over multiple generations in the past 20 years. Due to the smaller pediatric market, less focus appears to have been placed on the development of the smaller 5 mm instruments. As pediatric urology continues to utilize robotic technology for minimally invasive surgery, there is hope that additional pediatric-friendly instruments and components will be developed, either by Intuitive Surgical or one of the new robotic platforms in development that are working to address many of the shortcomings of current systems. These new robotic platforms include improved haptic feedback systems, flexible scopes, easier maneuverability, and even adaptive machine learning concepts to bring robotic assisted laparoscopic surgery to the next level. In this report, we review the present and upcoming technological advances of the current Da Vinci surgical systems as well as various new robotic platforms, each offering a unique set of technological advantages. As technology progresses, the understanding of and access to these new robotic platforms will help guide pediatric urologists into the next forefront of minimally invasive surgery.

## Introduction

The introduction of laparoscopy for children with non-palpable testicles in the 1960s has led to widespread adoption within the field of pediatric urology, and even replaced open surgery in some situations as the gold standard (1). Although laparoscopy enabled smaller surgical scars and decreased hospital stays, widespread use in complex reconstructive cases did not occur due to limitations on surgeon dexterity with available laparoscopic instruments, visualization, and sensory feedback (2, 3). Specifically in pediatrics, the need for more delicate tissue handling and adaptation to a smaller operative working space posed a further challenge in minimally invasive surgery (4). The introduction of the da Vinci Surgical System (Intuitive Surgical, Inc., Sunnyvale, CA) in 1999 addressed many of the basics compromises encountered in laparoscopy (5). Some key components included the 4th arm for retraction, 3-D visualization, 7-degree range of motion and tremor elimination. Furthermore, with progressing editions (S, Si, Xi, and X), Intuitive Surgical has advanced the system's visualization and instrumentation, as well as its teaching capabilities with dual-console systems and skill simulators.

## Integration of the da Vinci System Into Pediatric Urology

Adaptation of the robot into adult urology first occurred for prostatectomy (6), and pediatric urology soon followed with the first robotic pyeloplasty (7). Pyeloplasty was first performed laparoscopically in adults in 1993 (8, 9) and in children in 1995 (10). In comparison to open surgery, laparoscopic pyeloplasty demonstrated better postoperative pain control and decreased length of hospital stays (11), but the intracorporeal suturing carried a steep learning curve, limiting widespread adaptation (2, 3). Compared to conventional laparoscopic pyeloplasty, robot-assisted laparoscopic pyeloplasty demonstrated a lower complication rate (OR 0.56, 95% CI 0.37–0.84, *p* = 0.005) and higher success rate (OR 2.76, 95% CI 1.30–5.88, *p* = 0.008) with reductions in average operative times of 27 mi (*p* = 0.003) and reductions in length of hospital stays of 1.2 days (*p* = 0.003) (12).

Despite these advantages, the higher associated costs of robotic surgery do remain a concern. In comparison to open pyeloplasty, costs of laparoscopic pyeloplasty were very similar, but robotic pyeloplasty increased the total median cost from $7,221 to $10,780 in a population-based study. For all approaches, operating room costs were the greatest contributor, but with robotic pyeloplasty, the supplies costs were also much higher (13). Another report showed an 1.2-day improvement in length of stay with robotic vs. open pyeloplasty, amounting to an average savings of parental wages of $90.01 and hospital expenses of $612.80 when excluding amortization robot costs. However, this benefit was lost when amortization costs were included (14). Similarly, Rowe et al. broke down robotic costs into direct costs for the individual case and indirect costs of robot purchase and maintenance, finding that the inclusion of only direct costs showed an 11.9% savings for robotic surgery mostly due to shorter length of stay. Since the inclusion of indirect robotic costs tip the scales in the other direction, they concluded that high surgical volume and potential competition could reduce overall robotic surgery costs (15).

Fortunately, the rate of robotic pyeloplasty has increased annually at a rate of 29%, accounting for 40% of all cases in the US in 2015 (13). However, in comparison to adult robotic volumes, the pediatric volumes are still quite low, deterring some children's hospitals from individually purchasing a robot. Each institution evaluates the pros and cons of purchasing a da Vinci system due to implied maintenance costs, and some have found creative solutions, such as purchasing the robotic system at the children's hospital but subsidizing costs by allowing adult surgeons to also perform robotic procedures in the children's hospital for a set fee per patient (16). While such alternatives help decrease to costs, it does not remove the other extra robotic costs due to the built-in obsolescence of the robotic instruments, which have a preset number of uses that are programmed into the memory chip of each instrument. This essential monopoly with higher costs provided by the da Vinci surgical system begs for alternative platforms that will bring competition to the market and ideally drive down prices (16).

In addition to cost, the use of the da Vinci surgical system in pediatric patients holds certain other concerns, especially related to the smaller patient size and working space in young children and infants (4). With surgeon experience, tricks to maximize the smaller working space have been developed, such as a more linear, less triangulated trocar placement, delicate cushioning to protect the patient, and intussusception of trocars during placement to prevent injuries (17). Furthermore, careful padding, and port placement to avoid collisions are critical to protect small pediatric patients where the robotic arms are sometimes larger than the patient's body (18). Keeping these nuances and complexities in mind, infant robotic pyeloplasty cases have been performed with similar outcomes (19, 20). Thus, the utilization of robotic technology has grown in pediatric urology, and likely will continued to do so in the future to potentially even become the gold standard for certain reconstructive cases (21).

After pyeloplasty, the robotic platform has been applied to other reconstructive procedures, including extravesical ureteral reimplantation (22), appendicovesicostomy (23), and even bladder augmentation (24). It remains unclear if robotic ureteral reimplantation can provide superior, or even equivalent outcomes to the open correlate due to the high success rates of open surgery, but no significant differences in success rates or complication rates were seen in a recent multi-institutional study after the initial 30-case learning curve (22). The more complex reconstructive procedures still require further studies to determine the benefits of robotic assistance for these cases.

In addition to progressive technologic advances in the da Vinci robotic system in its evolving generations, incorporation of robotic technology with single site surgery has led to robot-assisted laparoendoscopic single site surgery (LESS) to allow for surgery to be performed through a single albeit slightly longer incision. This technique has shown success in laparoscopic surgery for extirpative procedures, such as nephrectomy (25, 26), but no reports of robot-assisted LESS in pediatric patients have been published to date. It is possible that the adaptation of the newer robotic platforms may lead to new opportunities in pediatric reconstruction. The da Vinci surgical robot can be combined with Intuitive Surgical's own single-site port platform or with other port platforms, including GelPoint (Applied Medical, Rando Santa Margarita, CA (27). Recent reports of the da Vinci single-site platform for donor nephrectomy noted that the procedure was safe, but without any clear tangible benefit (28). Again, this serves as one example of the need for better articulating instruments and energy sources that could be the key for expanding robotic technology to single site surgery on a larger scale. The most recent da Vinci SP platform is compatible with the Xi system and has an articulating endoscope with up to three fully-wristed, elbowed instruments, all through a single 2.5 cm port (29). While this device shows promise for use in pediatric urology, no such reports have yet been published. One immediate criticism of the device is the amount of working space needed internally to allow the usage and articulation of the instruments. Thus, while single-port robotic surgery is on the horizon, it is not yet been adapted in the field of pediatric urology.

Annually the Da Vinci Surgical robot is used to perform more than 750,000 procedures world-wide (30), but there remains vast areas for technological improvements, especially for pediatric patients. Smaller working spaces restrict surgeon dexterity and ability to perform task with the robot. One study noted that no surgical task could be performed in a space smaller than a 40 mm cubic box due to severe external robotic arm collisions (31). In smaller patients, 5 mm instruments offer the advantage of a smaller diameter incisions and finer needle forces for tissue handling (32). However, while there is a large variety of instruments available in the 8 mm size, only a limited selection is available in the 5 mm that would be better suited for children. While these limited number of 5-mm instruments are sufficient to successfully perform a pediatric robotic pyeloplasty (33), the limited selection of instruments has led many pediatric surgical specialists to use the 8 mm instruments despite its larger sizes, especially when the robot is shared with adult urology colleagues. Furthermore, use of a 5 mm lens removes the advantageous 3-D image and the 5 mm instruments require more working space due to typical joint kinematics (31, 34). On the other hand, the da Vinci 8 mm instruments require less space for articulation and in a head-on comparison the 8 mm robotic instruments demonstrated better efficacy and safety in smaller workspaces (35). It is possible that better 5 mm instruments with the same articulation abilities of the 8 mm instruments would overcome this hurdle, however at present such options are not available from Intuitive Surgical. Unfortunately, with a smaller pediatric market and limited profit potential, the business case often keeps manufacturers from devoting resources and time toward the development of further pediatric-sized instruments.

Lastly, the da Vinci surgical system lacks haptic feedback which can pose some difficulty in both transitioning to and learning robotic surgery. With the advent of new robotic systems, there is hope for application of haptic technology, utilization of more and improved pediatric-sized instruments, and ideally a decrease in cost with increasing competition as many of the Da Vinci patents expire in 2019 (36).

## New Robotic Platforms

There are many different robotic platforms at various stages of development, and some are even commercially available. As of yet, none of these new technologies have been utilized in pediatric urology, but here we focus on the platforms that could potentially be useful in the field of pediatric urology specifically. Table 1 compares the various features available in the current da Vinci Surgical System and these new upcoming robotic platforms.

**Table 1:** Comparison of da Vinci surgical and new robotic systems.

**Company**	**Location**	**Robotic system**	**Approach**	**Status**	**Camera**	**Robotic segments**	**DOF**	**Haptic feedback**	**Additional features**
Intuitive surgical	Sunnyvale, CA	*Da Vinci Surgical System*	Laparoscopic LESS	Commercially available	2 HD-3D	4	7	None	Tremor filtration
TransEnterix	Morrisville, NC	*Senhance^*TM*^*	Laparoscopy	FDA anticipated	HD-3D (eye-tracking)	3	7	Present	Navigation, eye-tracking camera control system, individual robotic carts
Medrobotics Corp	Raynham, MA	*Flex ®*	Transoral	Commercially available	HD-2D (semirigid or flexible)	2	180°	None	Core flexible, steerable scope that becomes rigid once positioned
Cambridge Medical Robotics Ltd.	Cambridge, UK	*Versius*	Laparoscopic	FDA validation	HD-3D	Up to 5 (modular)	7	Present	Force and position measurements > 1000x/second, up to 5 arms, lightweight
Titan Medical Inc.	Toronto, ON	*SPORT^*TM*^*	LESS	FDA pending	HD-3D	1	Multiple	None	Singe incisions, multi-articulated instruments, single arm mobile cart
TransEnterix	Morrisville, NC	*SurgiBot^*TM*^*	LESS	FDA denied, marketing in China	HD-3D	2	6	None	Internal triangulation
German Aeurospace Center (DLR)	Oberpfaffenhogen-Weßling	*Mirosurge*	Laparoscopy	Commercially available (not US)	HD-3D	3–5	7	Present	Easy adaptation of MIRO arms
Medtronic	Minneapolis, MN	*Hugo*	Laparoscopic	Development	–	–	–	–	Flexible use—mass utilization to decrease cost
Nanyang Technological University	Singapore	*MASTER*	NOTES	Clinical Trial	2D endoscope	2	9	Present	For NOTES allows smaller instruments with larger forces, reconstruction navigation
BIOTRONIK	Berlin, Germany	*ViaCath*	NOTES	Commercially available (not US)	N/A	1	9	None	Use in ureteroscopy and endovascular procedures
Memic Innovative Surgery	Israel	*Hominis^*TM*^*	Laparoscopic LESS NOTES	Development	–	Humanoid shaped arms	360°	–	Humanoid shaped robotics arms
Virtual Incision and CAST (Omaha, NA)	Omaha, NA	*Miniature in vivo robot (MIVR)*	Advanced	Development	HD—flexible tip	2	6	None	Miniaturized unit artificial intelligence + machine learning
J&J/Alphabet	Mountain View, CA	*Verb Surgical*	Advanced	Development	–	–	–	-	“Surgery 4.0”—digital surgery combining robotics with data-driven machine learning

### Senhance Surgical Robotic System

An Italian company named Sofar first developed the ALF-X system that was later renamed to Senhance Surgical Robotic System (TransEnterix, Morrisville, NC) after being purchased by this US-based company. In October 2017, the FDA approved the system for both gynecologic and colorectal procedures (37). Both safe and successful outcomes in human subjects undergoing hysterectomy (38–41) and colorectal surgery (42) have been described in the literature. However, within the field of urology, only porcine studies have been previously reported (43).

The key components of this system include the “cockpit” that serves as a remote-control station unit, up to 4 manipulator arms, and HD-3D-technology camera, as well as a connection node. Instead of the bulky single cart used by the Da Vinci system, the Senhance system robotic arms each have their own individual carts, allowing for easy maneuverability. Furthermore, the use of magnets to attach the instruments to the individual robotic arm carts enables more rapid exchanges intraoperatively. All instruments are compatible with a 5 mm port except the camera and articulating needle holder, which require a 10 mm port (44). Unlike the Da Vinci robot, no articulating cutting tool is currently available, with plans for future development (45). In addition to carrying the same 7 degrees of freedom currently available in other systems, all of these robotic arms use haptic sensing to enhance surgical dissection and suturing. The haptic feedback includes 1:1 scaled force feedback, tissue consistency perception and translation of instrument stress. The surgeon controls the robotic arms and eye-tracking camera from the cockpit, which includes comfortable ergonomic positioning (44). By tracking the surgeon's eye movements, the camera image is automatically centered to the surgeon's visual focus point and the amount of magnification can be adjusted by forward and backward head movements. The enhanced HD-3D-technology display is not only provided to the surgeon, but to the entire room. While this new system is promising, the system appears to have disadvantages currently when compared to other systems, including the use of large, bulky, and now multiple separate robotic arms, the need for polarizing glasses for the 3D-monitor eye tracking and the limited selection of articulating instruments (36, 46).

### Flex Robotic System

The Flex Robotic System, FDA-approved in July 2015, was developed by Medrobotics Corporation (Raynham, MA) for transoral robotic surgery (47). The system is comprised of a single-port operator-controlled flexible endoscope. The endoscope is guided by an outer robotic joystick with a touchscreen and magnified HD 2D visual display. An inner and outer segment with a single articulation point between the two comprises the endoscope, which through this mechanism, either can be semi-rigid or flexible, enabling passage of flexible instruments. Two endoscopic lumens provide a path for both fluid irrigation and electrical wiring. In addition, flexible instruments with 180-degree articulation and as small as 3 mm in size can be passed through the two External Accessory Channels (EAC).

In the world of oral surgery, the system has successfully and safely been used for the removal of lesions in the supraglottic larynx, hypopharynx, and oropharynx in human subjects (48–50). Based on these promising outcomes, in January 2018, the use of the Flex Robotic system was approved for other procedures including thoracic, gynecologic, and general surgical procedures in the thorax and abdomen via skin incisions rather than natural orifices (51). Due to the advantageous ease of setup and transport along with the smaller surgical footprint, it is possible that the expanded FDA approval may lead to more widespread use.

### Versius Robotic System

CMR Surgical (Cambridge, UK) has developed a new surgical robotic system, Versius, that recently launched its first U.S. training program in partnership with Florida Hospital Nicholson Center. The system features a lightweight robot for transabdominal surgeries, including general, colorectal, gynecologic, and urologic procedures. After successful cadaveric trials for electrocautery, needle driving, tissue handling, and suturing, the company proceeded to 9 weeks of FDA validation studies in Florida with plans for a U.S. launch in 2019 (52). This anticipated introduction may lead to a worthwhile competing system to the current Da Vinci robotic system.

Through a modular design, the system offers diversity and flexibility when it comes to operating room positioning. Up to 5 different robotic arms can be used with several available 5 mm instruments, including electrocautery electrodes, needle drivers, graspers, and scissors (53). Joystick controllers at the robotic console are used to manipulate the modular wristed robotic arms, and the console monitor can be visualized with HD-3D glasses. Furthermore, the robotic arms can transmit haptic feedback with force and position measurements occurring 1,000 times per second (46).

### SPORT™ Surgical System

Through integration of the LESS approach to a console-based platform, a Toronto-based company has created the Single Port Orifice Robotic Technology (SPORT) Surgical System (Titan Medical Inc.). The design utilizes multi-articulated instruments with disposable and replaceable tips. For the single port, the incision can be as small as 2.5 cm, and via this port, the entire collapsible system can be placed intracorporeally (54). The ergonomic open workstation includes a variety of hand controllers, foot pedals, and a 3D HD flat touchscreen monitor. With the single port orifice, only one single-arm mobile patient cart is needed, thus improving the ease of use. Animal models for single port nephrectomy have shown significant success to date (36) and in 2019, an application for FDA approval is anticipated (55).

### SurgiBot™

TransEnterix (Morrisville, NC) is also developing another surgical robotic system named the SurgiBot^TM^ specifically for underserved populations by requiring a minimal acquisition investment. All flexible instruments are placed through a single channel in a single-incision site (46). Similar to many other robotic platforms, the SurgiBot includes 3D vision, ergonomic control with internal triangulation, and precision movement with scaling-incision site via a single channel. The pre-clinical trials at Baptist Health Medical Group in Miami consisted of two cholecystectomy and two nephrectomy procedures in a porcine model in 2015 (56). Thereafter, the platform did not receive FDA approval in 2016 as it failed to show equivalence to devices on the market. Since then, TransEnterix transferred the ownership of SurgiBot System assets to Great Belief International Limited with the option to distribute the product outside of China. The future of SurgiBot remains yet to be seen (57).

### MiroSurge

In Oberpfaffenhofen-Weßling, the Robotics and Medtronics Center (MDR) of the German Aerospace Center (DLR) is developing a telemanipulated minimally invasive robotic surgery (MIRS) system named MiroSurge. Individual minimally invasive robot-assisted (MIRO) arms carrying an instrument can be mounted to the bed rails at various locations (58–60). Anywhere from three to five MIROs can be used with two guiding instruments by left and right manipulation and one for the endoscopic camera (61). Not only does each MIRO arm carry seven DOF with haptic feedback, but it can also adapt to various uses, such as actuated surgical instrumentation [Tobergte; (62)].

The MIRO arms have joints with torque and position sensors, which enable manual shifting and positioning of the arms. In impedance-controlled mode the insertion points are planned preoperatively based on algorithms specific for the robot's kinematics (61). Thus, far the system has been used for endoscopic teleoperated minimally invasive and open abdominal and thoracic surgeries. While it has not been officially announced, there is speculation that the MicroSurge technology has been licensed for use, although FDA approval information is not yet available (60).

### Medtronic Robotic Surgery Program

The Minimally Invasive Therapies Group at Medtronic (Minneapolis, MN) has been working to develop a robotic platform for which a name has not been officially released. Previously the name Einstein had been mentioned (46), and now there are rumors that the surgical robot will be named Hugo (63). The development has occurred through multiple partnerships with Mazor Robotics, the German Aerospace Center (DLR) and Covidien. Many of the details for this system have not been revealed, but the platform has been under development for more than 6 years and is already past its tenth prototype. The system is advertised to be flexible with a wide range of uses in bariatric, thoracic, colorectal, general, and urologic surgeries. In this fashion they hope to decrease costs by enhanced utilization of the robotic technology (64). Per interviews with Medtronic, the system has been trialed by many surgeons and they anticipated an initial system launch in India (65). However, delays in the initial launch, now aimed to be by end of fiscal year 2019, have led to some drops in the Medtronic stock (66, 67).

### Master

Natural orifice transluminal endoscopic surgery (NOTES) takes LESS one step further, allowing an abdominal procedure to be performed through an internal incision in the stomach, vagina, bladder, or colon. However, many complicated procedures cannot be performed via conventional endoscopy and tools due to limited dexterity (68–70). The Master and Slave Transulminal Endoscopic Robotic (MASTER) allows for dexterity, triangulation, haptic feedback and a navigation system with real-time 3D reconstruction. opening the door to many new applications of NOTES (71). This platform created by Nanyang Technological University and National University Health System consists of an endoscope and two effector arms—a monopolar hook cautery and graspers. The surgeon operates the effector arms, which can be bimanually steered through a master control device while an endoscopist guides the endoscope to the desire location, controlling suction and inflation (69). In comparison to other technologies the MASTER allows for smaller instruments with larger forces, but additional work is still planned to improve automated movements and haptic feedback (68). While human procedures have yet to be reported, the MASTER system has demonstrated initial success in animal models, specifically by performing endoscopic sub-mucosal dissections for segmental hepatectomies (69).

### ViaCath System

BIOTRONIK (Berlin, Germany) has developed another robotic platform for NOTES, the ViaCath system, with haptic feedback and interchangeable instruments, including graspers, scissors, electrocautery knife and needle holders (68). The surgeon at the console steers a standard colonoscope or endoscope with long-shafted instruments running alongside through an articulated flexible overtube (71). The instruments have better flexibility and decreased friction with the stainless steel and Teflon design, each with seven DOF along with the positioning arm (68). Furthermore, the overtube adds another two DOF via two joints, totaling nine DOF (72). Once the overtube is appropriately placed the two working instruments can be triangulated through a nose cone with cable-actuated gripper devices that allow rotary motion (68).

However, the system does lack appropriate spatial orientation with incomplete triangulation due to the parallel instrument orientations (71). Furthermore, the manipulation forces available are smaller than conventional laparoscopic instruments which could impede controlled device manipulation (72). While the robotic design can be catered toward NOTES, no such studies have yet been done. However, the system has been useful in endoscopic cases, such as ureteropyeloscopy on porcine models (73), and may present a new role for endoscopic procedures in pediatric urology.

### Hominis™ Surgical System

Memic Innovative Surgery designed the *Hominis*™ *Surgical System* robotic platform to emulate human dexterity through small humanoid-shaped robotic arms with a novel 360-degree articulation. The system not only allows for both multiport and single port approaches, but also provides a platform for transvaginal access to perform hysterectomy. The Hominis system may provide the potential for improved ergonomics, lower costs, smaller footprint and variability in access with what is described as “seamless” robotic surgery (74, 75). However, this company appears to be in its early stages with no human or animal studies reported as of yet. For a future FDA submission, they are in the process of evaluating usability review.

### Miniature *in vivo* Robot (MIVR)

Through a joint venture between The Center for Advanced Surgical Technology (CAST) at the University of Nebraska Medical Center in Omaha and Virtual Incision, a miniaturized *in vivo* robot (MIVR) was developed. This novel platform aims to reduce the robot size as well as improve intraperitoneal maneuverability to enable access to all four quadrants from a single umbilical entry point (76). The miniaturized robotic system is comprised of a novel surgeon-controlled flexible tip laparoscope and two robotic arms with multiple joints. The end effectors of the robotic arms can easily be changed and adapted for different operative needs and instruments. Additionally, the instrument movements are tracked and ultimately guided with a combination of artificial intelligence and machine learning technologies (77, 78). By localizing the drive technology within the small robotic arms, there is no need for larger platforms, further facilitating its use in the operating room (78). In the future, it is envisioned that the use of a combination of miniaturized robots simultaneously will cater to the specific complexity and needs of a particular procedure but with entry of all robots through the same entry site (78).

At present, successful use of a MIVR for colectomy was described in porcine studies (77). This same technology was applied to feasibility and safety human trials in South Africa, again showing successful outcomes for robotic colectomy (79). Further development of the platform is still in progress, with plans for small inexpensive robots for common routine procedures, such as cholecystectomy or hernia repair. Pending the finalization of these designs, an application for FDA approval is planned in the near future (76).

### Verb Surgical

A joint venture between Johnson & Johnson's medical device company Ethicon Endo-Surgery and Alphabet's (Google) Verily Life Sciences, led to the creation of Verb Surgical, Inc. (J&J/Alphabet, Mountain View, CA, USA) (80). This company is striving to create an autonomous surgical robot rather than just a surgeon controlled tool, which they envision as the next advance for the digital age (81). Although the company provided a demonstration to collaborators in January 2017 (80), little information has been released to date about anticipated next steps and plans.

Thus far, the device is said to “democratize surgery” with increased surgeon access to information through advancements in data analytics and machine learning, which they described as one step farther than the basic goals of robotic platforms of advanced visualization, instrumentation and connectivity (82). This new era of “digital surgery” has been coined as surgery 4.0, an advancement from the initial open surgery (1.0) to minimally invasive surgery (2.0) to initial robotic surgery (3.0) (83). Theoretically their prototype works to decrease costs and increase surgeon access through a combinations of robotic technology and data-driven machine learning (82).

### Future Directions

Examination of emerging robotic platforms has opened a vast array of possibilities for the future of robotic surgery. With these continued advancements, the trend appears to be moving toward less incisions down to a single port platform, and possibly even no incision in the future. Furthermore, the combination of virtual reality technology and robotic surgery may lead to a completely new era of surgery that may include autonomous robotic surgery in the future.

## Author Contributions

KS and CK drafted, revised, and approved the final manuscript.

### Conflict of Interest Statement

CK is a course director and consultant for Intuitive Surgical. The remaining author declares that the research was conducted in the absence of any commercial or financial relationships that could be construed as a potential conflict of interest.

## Abbreviations

3D: Three-dimensional
CI: Confidence Interval
DOF: Degrees of Freedom
FDA: Food and Drug Administration
HD: High Definition
LESS: Laparoendoscopic Single Site
NOTES: Natural Orifice Transluminal Endoscopic Surgery
OR: Odds Ratio.

## References

[1] Gobbi D Midrio P Gamba P. Instrumentation for minimally invasive surgery in pediatric urology. Transl Pediatr. (2016) 5:186–204. 10.21037/tp.2016.10.07 27867840 PMC5107385

[2] Chen RN Moore RG Kavoussi LR. Laparoscopic pyeloplasty. Indications, technique, and long-term outcome. Urol Clin North Am. (1998) 25:323–30. 10.1016/S.0094-0143(05)70021-5 9633588

[3] Bauer JJ Bishoff JT Moore RG Chen RN Iverson AJ Kavoussi LR. Laparoscopic versus open pyeloplasty: assessment of objective and subjective outcome. J Urol. (1999) 162(3 Pt 1):692–5. 10.1097/00005392-199909010-00016 10458344

[4] Jaffray B. Minimally invasive surgery. Arch Dis Child. (2005) 90:537–42. 10.1136/adc.2004.062760 15851444 PMC1720379

[5] Rassweiler JJ Teber D. Advances in laparoscopic surgery in urology. Nat Rev Urol. (2016) 13:387–99. 10.1038/nrurol.2016.70 27215426

[6] Rassweiler J Binder J Frede T. Robotic and telesurgery: will they change our future? Curr Opin Urol. (2001) 11:309–20. 10.1097/00042307-200105000-00012 11371786

[7] Atug F Woods M Burgess SV Castle EP Thomas R. Robotic assisted laparoscopic pyeloplasty in children. J Urol. (2005) 174(4 Pt 1):1440–2. 10.1097/01.ju.0000173131.64558.c9 16145459

[8] Kavoussi LR Peters CA. Laparoscopic pyeloplasty. J Urol. (1993) 150:1891–4. 10.1016/S0022-5347(17)35926-8 8230528

[9] Schuessler WW Grune MT Tecuanhuey LV Preminger GM. Laparoscopic dismembered pyeloplasty. J Urol. (1993) 150:1795–9. 10.1016/S0022-5347(17)35898-6 8230507

[10] Peters CA Schlussel RN Retik AB. Pediatric laparoscopic dismembered pyeloplasty. J Urol. (1995) 153:1962–5. 10.1016/S0022-5347(01)67378-6 7752371

[11] Klingler HC Remzi M Janetschek G Kratzik C Marberger MJ. Comparison of open versus laparoscopic pyeloplasty techniques in treatment of uretero-pelvic junction obstruction. Eur Urol. (2003) 44:340–5. 10.1016/S0302-2838(03)00297-5 12932933

[12] Light A Karthikeyan S Maruthan S Elhage O Danuser H Dasgupta P. Peri-operative outcomes and complications after laparoscopic vs robot-assisted dismembered pyeloplasty: a systematic review and meta-analysis. BJU Int. (2018) 122:181–94. 10.1111/bju.14170 29453902

[13] Varda BK Wang Y Chung BI Lee RS Kurtz MP Nelson CP Has the robot caught up? National trends in utilization, perioperative outcomes, and cost for open, laparoscopic, and robotic pediatric pyeloplasty in the United States from 2003 to 2015. J Pediatr Urol. (2018) 336:e1–336.e8. 10.1016/j.jpurol.2017.12.010 PMC6105565 29530407

[14] Behan JW Kim SS Dorey F De Filippo RE Chang AY Hardy BE. Human capital gains associated with robotic assisted laparoscopic pyeloplasty in children compared to open pyeloplasty. J Urol. (2011) 186:1663–7. 10.1016/j.juro.2011.04.019 21862079

[15] Rowe CK Pierce MW Tecci KC Houck CS Mandell J Retik AB. A comparative direct cost analysis of pediatric urologic robot-assisted laparoscopic surgery versus open surgery: could robot-assisted surgery be less expensive? J Endourol. (2012) 26:871–7. 10.1089/end.2011.0584 22283146

[16] Steyaert H Van Der Veken E Joyeux L. Implementation of robotic surgery in a pediatric hospital: lessons learned. J Laparoendosc Adv Surg Tech A. (2019) 29:136–40. 10.1089/lap.2018.0426 30222503

[17] Howe A Kozel Z Palmer L. Robotic surgery in pediatric urology. Asian J Urol. (2017) 4:55–67. 10.1016/j.ajur.2016.06.002 29264208 PMC5730905

[18] Gundeti MS Kojima Y Haga N Kiriluk K. Robotic-assisted laparoscopic reconstructive surgery in the lower urinary tract. Curr Urol Rep. (2013) 14:333–41. 10.1007/s11934-013-0328-7 23740381

[19] Ballouhey Q Villemagne T Cros J Szwarc C Braik K Longis B A comparison of robotic surgery in children weighing above and below 15.0 kg: size does not affect surgery success. Surg Endosc. (2015) 29:2643–50. 10.1007/s00464-014-3982-z 25480612

[20] Baek M Au J Huang GO Koh CJ. Robot-assisted laparoscopic pyeloureterostomy in infants with duplex systems and upper pole hydronephrosis: variations in double-J ureteral stenting techniques. J Pediatr Urol. (2017) 13:219–20. 10.1016/j.jpurol.2016.12.008 28153776

[21] Andolfi C Kumar R Boysen WR Gundeti MS. Current status of robotic surgery in pediatric urology. J Laparoendosc Adv Surg Tech A. (2019) 29:159–66. 10.1089/lap.2018.0745 30592689

[22] Boysen WR Akhavan A Ko J Ellison JS Lendvay TS Huang J Prospective multicenter study on robot-assisted laparoscopic extravesical ureteral reimplantation (RALUR-EV): outcomes and complications. J Pediatr Urol. (2018) 14:265–66. 10.1016/j.jpurol.2018.03.004 29503220

[23] Gundeti MS Petravick ME Pariser JJ Pearce SM Anderson BB Grimsby GM. A multi-institutional study of perioperative and functional outcomes for pediatric robotic-assisted laparoscopic Mitrofanoff appendicovesicostomy. J Pediatr Urol. (2016) 12:386.e381–386.e385. 10.1016/j.jpurol.2016.05.031 27349147

[24] Murthy P Cohn JA Selig RB Gundeti MS. Robot-assisted laparoscopic augmentation ileocystoplasty and mitrofanoff appendicovesicostomy in children: updated interim results. Eur Urol. (2015) 68:1069–75. 10.1016/j.eururo.2015.05.047 26187785

[25] Kawauchi A Naitoh Y Miki T. Laparoendoscopic single-site surgery for pediatric patients in urology. Curr Opin Urol. (2011) 21:303–8. 10.1097/MOU.0b013e3283468d40 21499105

[26] Luithle T Szavay P Fuchs J. Single-incision laparoscopic nephroureterectomy in children of all age groups. J Pediatr Surg. (2013) 48:1142–6. 10.1016/j.jpedsurg.2013.01.040 23701796

[27] Kan HC Pang ST Wu CT Chang YH Liu CY Chuang CK. Robot-assisted laparoendoscopic single site adrenalectomy: a comparison of 3 different port platforms with 3 case reports. Medicine. (2017) 96:e9479. 10.1097/MD.0000000000009479 29390591 PMC5758293

[28] LaMattina JC Alvarez-Casas J Lu I Powell JM Sultan S Phelan MW. Robotic-assisted single-port donor nephrectomy using the da Vinci single-site platform. J Surg Res. (2018) 222:34–8. 10.1016/j.jss.2017.09.049 29273373

[29] Intuitive Surgical Da Vinci SP: For Narros Access Surgery. (2019). Available online at: https://www.intuitive.com/en-us/products-and-services/da-vinci/systems/sp (accessed May 2, 2019).

[30] Cole AP Trinh QD Sood A Menon M. The rise of robotic surgery in the new millennium. J Urol. (2017) 197:S213–5. 10.1016/j.juro.2016.11.030 28010986

[31] Thakre AA Bailly Y Sun LW Van Meer F Yeung CK. Is smaller workspace a limitation for robot performance in laparoscopy? J Urol. (2008) 179:1138–1142; discussion 1142–1133. 10.1016/j.juro.2007.10.091 18206956

[32] Ballouhey Q Villemagne T Cros J Vacquerie V Berenguer D Braik K. Assessment of paediatric thoracic robotic surgery. Interact Cardiovasc Thorac Surg. (2015) 20:300–3. 10.1093/icvts/ivu406 25476460

[33] Paradise HJ Huang GO Elizondo Saenz RA Baek M Koh CJ. Robot-assisted laparoscopic pyeloplasty in infants using 5-mm instruments. J Pediatr Urol. (2017) 13:221–2. 10.1016/j.jpurol.2016.12.011 28153777

[34] Pelizzo G Nakib G Romano P Avolio L Mencherini S Zambaiti E. Five millimetre-instruments in paediatric robotic surgery: advantages and shortcomings. Minim Invasive Ther Allied Technol. (2015) 24:148–53. 10.3109/13645706.2014.975135 25363461

[35] Ballouhey Q Clermidi P Cros J Grosos C Rosa-Arsene C Bahans C. Comparison of 8 and 5 mm robotic instruments in small cavities: 5 or 8 mm robotic instruments for small cavities? Surg Endosc. (2018) 32:1027–34. 10.1007/s00464-017-5781-9 28840328

[36] Rassweiler JJ Autorino R Klein J Mottrie A Goezen AS Stolzenburg JU. Future of robotic surgery in urology. BJU Int. (2017) 120:822–41. 10.1111/bju.13851 28319324

[37] U. S. Food and Drug FDA Clears New Robotically-Assisted Surgical Device For Adult Patients. (2017). Available online at: http://news.doximity.com/entries/9699292?authenticated=false (accessed March 26, 2018).

[38] Fanfani F Restaino S Gueli Alletti S Fagotti A Monterossi G Rossitto C. TELELAP ALF-X robotic-assisted laparoscopic hysterectomy: feasibility and perioperative outcomes. J Minim Invasive Gynecol. (2015) 22:1011–7. 10.1016/j.jmig.2015.05.004 25982854

[39] Fanfani F Monterossi G Fagotti A Rossitto C Gueli Alletti S Costantini B. The new robotic TELELAP ALF-X in gynecological surgery: single-center experience. Surg Endosc. (2016) 30:215–21. 10.1007/s00464-015-4187-9 25840895

[40] Fanfani F Restaino S Rossitto C Gueli Alletti S Costantini B Monterossi G. Total Laparoscopic (S-LPS) versus TELELAP ALF-X robotic-assisted hysterectomy: a case-control study. J Minim Invasive Gynecol. (2016) 23:933–8. 10.1016/j.jmig.2016.05.008 27247263

[41] Gueli Alletti S Rossitto C Cianci S Restaino S Costantini B Fanfani F. Telelap ALF-X vs standard laparoscopy for the treatment of early-stage endometrial cancer: a single-institution retrospective cohort study. J Minim Invasive Gynecol. (2016) 23:378–83. 10.1016/j.jmig.2015.11.006 26602025

[42] Spinelli A David G Gidaro S Carvello M Sacchi M Montorsi M First experience in colorectal surgery with a new robotic platform with haptic feedback. Colorectal Disease. (2018) 20:228–35. 10.1111/codi.13882 28905524

[43] Bozzini G Gidaro S Taverna G. Robot-assisted laparoscopic partial nephrectomy with the ALF-X Robot on Pig models. Eur Urol. (2016) 69:376–7. 10.1016/j.eururo.2015.08.031 26361168

[44] TranEnterix Senhance™. (2018). Available online at: https://www.transenterix.com/overview/ (accessed March 26, 2018).

[45] Rao PP. Robotic surgery: new robots and finally some real competition! World J Urol. (2018) 36:537–41. 10.1007/s00345-018-2213-y 29427003

[46] Peters BS Armijo PR Krause C Choudhury SA Oleynikov D. Review of emerging surgical robotic technology. Surg Endosc. (2018) 32:1636–55. 10.1007/s00464-018-6079-2 29442240

[47] Medrobotics Flex® Robotic System: Expanding the Reach of Surgery®. (2018). Available online at: https://medrobotics.com/gateway/flex-system-int/ (accessed March 26, 2018).

[48] Lang S Mattheis S Hasskamp P Lawson G Guldner C Mandapathil M. A european multicenter study evaluating the flex robotic system in transoral robotic surgery. Laryngoscope. (2017) 127:391–5. 10.1002/lary.26358 27783427

[49] Mattheis S Hasskamp P Holtmann L Schafer C Geisthoff U Dominas N. Flex robotic system in transoral robotic surgery: The first 40 patients. Head Neck. (2017) 39:471–5. 10.1002/hed.24611 27792258

[50] Tan Wen Sheng B Wong P Teo Ee Hoon C. Transoral robotic excision of laryngeal papillomas with Flex(R) Robotic System - A novel surgical approach. Am J Otolaryngol. (2018) 355–8. 10.1016/j.amjoto.2018.03.011 29525139

[51] Taylor NP FDA Clears Medrobotics' Robotic Surgical Platform for Expanded Use. (2018). Available online at: https://www.fiercebiotech.com/medtech/fda-clears-medrobotics-robotic-surgical-platform-for-expanded-use (accessed March 26, 2018).

[52] CMR Surgical CMR Reveals Versius Robotic Surgery System. (2016). Available online at: https://cmrsurgical.com/cmr-reveals-versius-robotic-surgery-system/ (accessed March 26, 2018).

[53] Ellis R UK Scientists Create World's Smallest Surgical Robot to Start A Hospital Revolution. (2017). Available online at: https://www.theguardian.com/society/2017/aug/19/worlds-smallest-surgical-robot-versius-keyhole-hospital-revolution?CMP=share_btn_link (accessed March 26, 2018).

[54] Titan Medical Inc SPORT™ Surgical System. (2018). Available online at: https://titanmedicalinc.com/technology/ (accessed March 26, 2018).

[55] Idrus AA On Track for 2019 Launch, Titan Medical Installs Its First Surgical Robot in Florida (2017). Available online at: https://www.fiercebiotech.com/medtech/track-for-2019-launch-titan-medical-installs-its-first-surgical-robot-florida (accessed March 26, 2018).

[56] Haskins O TransEnterix Completes SurgiBot Pre-Clinical FDA Work. (2015). Available online at: http://www.bariatricnews.net/?q=node/1856 (accessed May 15, 2019).

[57] Ohnesorge L TransEnterix Announces Global SurgiBot System Agreement. (2017). Available online at: https://www.businesswire.com/news/home/20171218005245/en/TransEnterix-Announces-Global-SurgiBot-System-Agreement (accessed May 15, 2019).

[58] Hagn U Konietschke R Tobergte A Nickl M Jorg S Kubler B. DLR MiroSurge: a versatile system for research in endoscopic telesurgery. Int J Comput Assist Radiol Surg. (2010) 5:183–93. 10.1007/s11548-009-0372-4 20033517

[59] Beasley RA Medical robots: current systems and research directions. J Robot. (2012) 2012:401613 10.1155/2012/401613

[60] Henry BN Santo G Peering Behind the Veil of Secrecy in Surgical Robotics & 2016 Market Outlook. RBC Capital Markets (2017).

[61] Konietschke R Hagn U Nickl M Jorg S Tobergte A Passig G The DLR MiroSurge - A robotic system for surgery. In: 2009 IEEE International Conference on Robotics and Automation. Kobe (2009). p. 1589–90. 10.1109/ROBOT.2009.5152361

[62] Tobergte AH Thielmann P Grange S Albu-Schaffer S Conti A Hirzinger F The sigma.7 haptick interface fo MiroSurge: a new bi-manual surgical console. In: 2011 IEEE/RSJ International Conference onf Intelligent Robots and Systems, San Francisco, CA (2001), p. 3023–30.

[63] Medtechy[y] Did Medtronic Accidentally Release the Name of Their Robotic Surgery Program? (2017). Available online at: https://www.medtechy.com/articles/2017/did-medtronic-accidentally-release-the-name-of-their-robotic-surgery-program (accessed May 15, 2019).

[64] Thibault M Finally, Details on Medtronic's Robotics Platform. (2016). Available online at: https://www.mddionline.com/finally-details-medtronics-robotics-platform (accessed May 5, 2019).

[65] Medtechy[y] Medtronic CEO Provides Updated on Robotic Surgery Program. (2019). Available online at: https://www.medtechy.com/articles/2019/medtronic-ceo-provides-update-on-robotic-surgery-program (accessed May 15, 2019).

[66] Gatlin A Medtronic Sinks After Its Rival To Intuitive Surgical Delayed. (2018). Available online at: https://www.investors.com/news/technology/medtronic-rises-after-topping-quarterly-expectations/ (accessed May 15, 2019).

[67] Perriello B UPDATE: Robotics Delay Pushes Medtronic Down Despite Fiscal Q3 Beats. (2018). Available online at: https://www.massdevice.com/medtronics-q3-beats-expectations/ (accessed May 15, 2019).

[68] Yeung BP Gourlay T. A technical review of flexible endoscopic multitasking platforms. Int J Surg. (2012) 10:345–54. 10.1016/j.ijsu.2012.05.009 22641123

[69] Lomanto D Wijerathne S Ho LK Phee LS. Flexible endoscopic robot. Minim Invasive Ther Allied Technol. (2015) 24:37–44. 10.3109/13645706.2014.996163 25627436

[70] Kume K. Flexible robotic endoscopy: current and original devices. Comput Assist Surg. (2016) 21:150–9. 10.1080/24699322.2016.1242654 27973963

[71] Klibansky D Rothstein RI. Robotics in endoscopy. Curr Opin Gastroenterol. (2012) 28:477–82. 10.1097/MOG.0b013e328356ac5e 22885946

[72] Prendergast JM Rentschler ME. Towards autonomous motion control in minimally invasive robotic surgery. Exp Rev Med Devices. (2016) 13:741–8. 10.1080/17434440.2016.1205482 27376789

[73] Aron M Haber GP Desai MM Gill IS. Flexible robotics: a new paradigm. Curr Opin Urol. (2007) 17:151–5. 10.1097/MOU.0b013e3280e126ab 17414511

[74] Memic Innovation Surgery Hominis - Robotic Surgery Made Natural. (2017). Available online at: https://www.kenes-exhibitions.com/old/biomed2016/wp-content/uploads/2016/05/MEMIC.docx (accessed March 26, 2018).

[75] Memic Hominis™- the Smallest, Farthest Reaching Surgical Robot. (2018). Available online at: https://www.memicmed.com/ (accessed March 26, 2018).

[76] Keenan J Virtual Incision Reels in $18M in Series B Round To Support Its Surgical Robotics. (2017). Available online at: https://www.fiercebiotech.com/medtech/virtual-incision-reels-18m-series-b-round-to-support-its-surgical-robotics (accessed March 26, 2018).

[77] Wortman TD Design, Analysis, and Testing of in vivo Surgical Robots. (2011). Department of Mechanical Engineering, University of Nebraska—Lincoln. Available online at: https://digitalcommons.unl.edu/mechengdiss/28/ (accessed March 26, 2018).

[78] Virtual Incision World's First Use of Miniaturized Robot in Human Surgery. (2016). Available online at: https://www.virtualincision.com/fim-surgery/ (accessed March 26, 2016).

[79] Bedem LJ Realization of a Demonstrator Slave for Robotic Minimally Invasive Surgery. Eindhoven: Technische Universiteit Eindhoven (2010).

[80] Verb Surgical Inc Verb Surgical Delivers Digital Surgery Prototype Demonstration to Collaboration Partners. (2017). Available online at: https://www.prnewswire.com/news-releases/verb-surgical-delivers-digital-surgery-prototype-demonstration-to-collaboration-partners-300397192.html (accessed March 26, 2018).

[81] Simonite T The Recipe for the Perfect Robot Surgeon. (2016). Available online at: https://www.technologyreview.com/s/602595/the-recipe-for-the-perfect-robot-surgeon/ (accessed March 26, 2018).

[82] Thibault M Here's the Latest From Verb Surgical. (2016). Available online at: https://www.mddionline.com/heres-latest-verb-surgical (accessed March 26, 2018).

[83] Khateeb OM Democratizing Surgery Part 1: What Verb Surgical is Creating. (2016). Available online at: https://www.linkedin.com/pulse/democratizing-surgery-how-verb-surgical-invented-new-category (accessed March 26, 2018).

